# The MentDis_ICF65+ study protocol: prevalence, 1-year incidence and symptom severity of mental disorders in the elderly and their relationship to impairment, functioning (ICF) and service utilisation

**DOI:** 10.1186/1471-244X-13-62

**Published:** 2013-02-18

**Authors:** Sylke Andreas, Martin Härter, Jana Volkert, Maria Hausberg, Susanne Sehner, Karl Wegscheider, Sven Rabung, Berta Ausín, Alessandra Canuto, Chiara Da Ronch, Luigi Grassi, Yael Hershkovitz, Paul Lelliott, Manuel Muñoz, Alan Quirk, Ora Rotenstein, Ana Belén Santos-Olmo, Arieh Shalev, Jens Siegert, Kerstin Weber, Hans-Ulrich Wittchen, Uwe Koch, Holger Schulz

**Affiliations:** 1 Department of Medical Psychology, Centre for Psychosocial Medicine, University Medical Centre Hamburg-Eppendorf, Martinistr. 52, Building W 26, Hamburg D-20246, Germany; 2 Institute for Psychology, Alpen-Adria Universität Klagenfurt, Klagenfurt A-9020, Austria; 3 Department of Biometry and Epidemiology, University Medical Centre Hamburg-Eppendorf, Martinistr. 52, Building W 34, Hamburg D-20246, Germany; 4 School of Psychology, University Complutense of Madrid, Campus de Somosaguas s/n, Madrid 28223, Spain; 5 Division of liaison psychiatry and crisis intervention, Department of psychiatry and mental health, University Hospitals of Geneva (HUG), Rue Gabrielle Perret-Gentil 4, Geneva 14 1211, Switzerland; 6 Section of Psychiatry, Department of Biomedical and Specialty Surgical Sciences, Corso Giovecca 203, Ferrara 44121, Italy; 7 Department of Psychiatry, Hadassah University Medical Center, Kiryat Hadassah, P.O.B 12000, Jerusalem 91120, Israel; 8 Royal College of Psychiatry, Mansell Street 21, E18AA, London, United Kingdom; 9 Institute of Clinical Psychology and Psychotherapy, Chemnitzer Straße 46, Dresden 01187, Germany

**Keywords:** Mental health, Mental disorders, Elderly, Prevalence, Incidence, Health care use, ICF, Epidemiology

## Abstract

**Background:**

The EU currently lacks reliable data on the prevalence and incidence of mental disorders in older people. Despite the availability of several national and international epidemiological studies, the size and burden of mental disorders in the elderly remain unclear due to various reasons. Therefore, the aims of the MentDis_ICF65+ study are (1) to adapt existing assessment instruments, and (2) to collect data on the prevalence, the incidence, and the natural course and prognosis of mental disorders in the elderly.

**Method/design:**

Using a cross-sectional and prospective longitudinal design, this multi-centre study from six European countries and associated states (Germany, Great Britain, Israel, Italy, Spain, and Switzerland) is based on age-stratified, random samples of elderly people living in the community. The study program consists of three phases: (1) a methodological phase devoted primarily to the adaptation of age- and gender-specific assessment tools for older people (e.g., the Composite International Diagnostic Interview, CIDI) as well as psychometric evaluations including translation, back translation; (2) a baseline community study in all participating countries to assess the lifetime, 12 month and 1 month prevalence and comorbidity of mental disorders, including prior course, quality of life, health care utilization and helpseeking, impairments and participation and, (3) a 12 month follow-up of all baseline participants to monitor course and outcome as well as examine predictors.

**Discussion:**

The study is an essential step forward towards the further development and improvement of harmonised instruments for the assessment of mental disorders as well as the evaluation of activity impairment and participation in older adults. This study will also facilitate the comparison of cross-cultural results. These results will have bearing on mental health care in the EU and will offer a starting point for necessary structural changes to be initiated for mental health care policy at the level of mental health care politics.

## Background

Mental health in the elderly has become increasingly important in recent years. According to the Eurostat 2011-based population projections, the number of people aged 65 and over is expected to rise by approximately 81% between 2010 and 2060 [1]. Older age is associated with an increased frequency of disease, the need for additional care and services, and leads to rising costs for health care systems [2]. Furthermore, while society’s improved ability to treat diseases and chronic conditions is a great achievement, advances in health care have also led to increased prevalence of many diseases in the elderly [3]. To keep pace with this continuing demographic shift, more information on morbidity rates in the elderly and particularly the size and burden of mental disorders is needed to optimise mental health care and to provide adequate services for elderly people.

There are two basic requirements towards this goal that are currently not given. First, a reliable and improved diagnostic instrument for the assessment and evaluation of mental disorders in the elderly is urgently needed. The standard instrument used for size and burden assessment in the community has major problems [4]. As evidenced by neuropsychological and cognitive science research [4] the typically long interview questions used in diagnostic interview lead to different patterns of information processing, that in return require older people to use heuristics that differ from younger adults. As a result, elderly respondents deny many symptom questions resulting in unreasonably low estimates for any disorders.

The second challenge is how to assess the functional impairments and disabilities resulting from psychopathology and mental disorders. In this respect the International Classification of Functioning, Disability and Health ICF [5], offers a good framework for understanding the health status of people with mental disorders.

### The international classification of functioning, disability and health (ICF)

**Figure 1:**
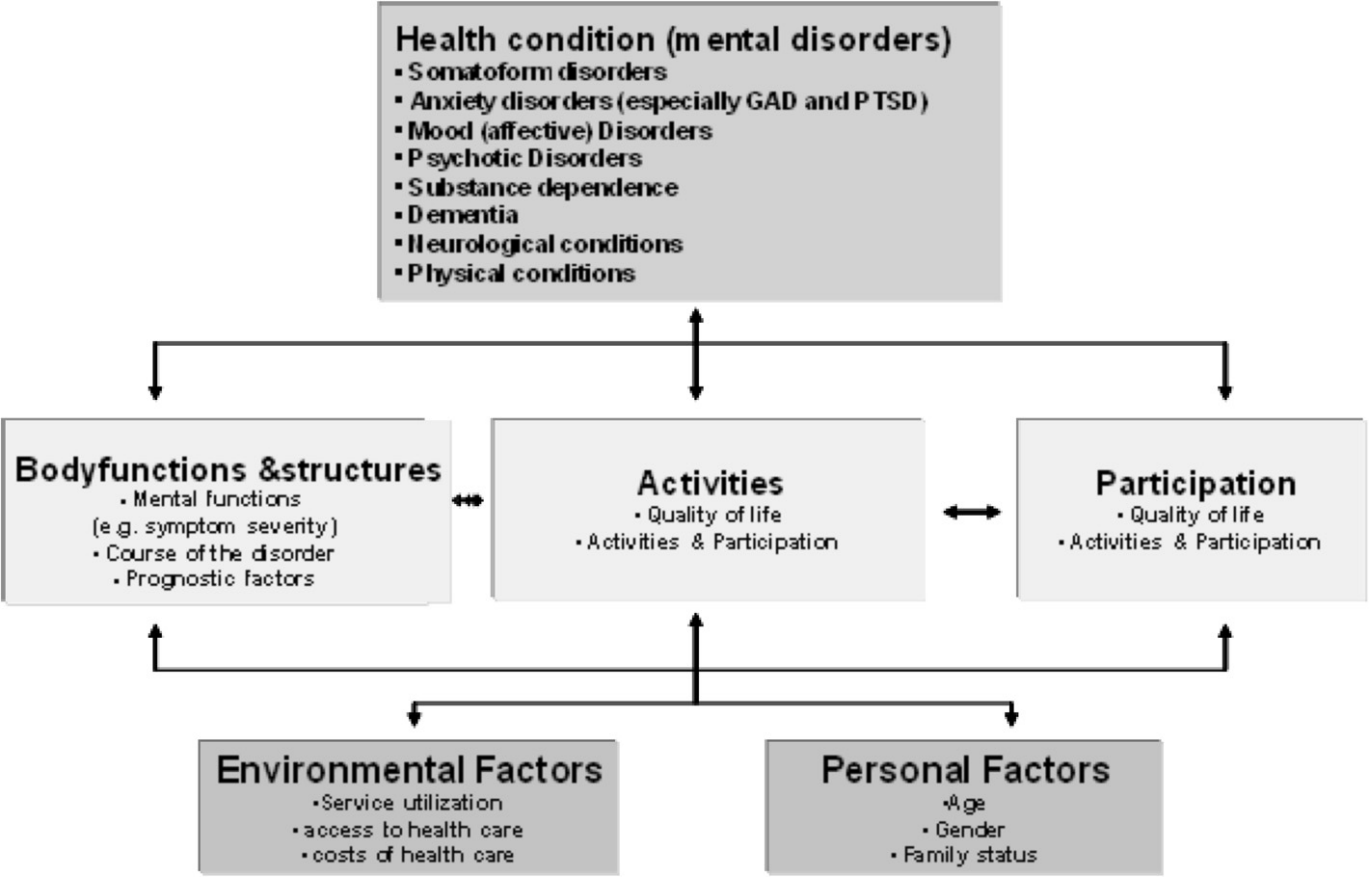
**The Model of the International Classification of Functioning, Disability and Health applied to the study aims of the MentDis_ICF65+ project (ICF) **[5]**.**

The International Classification of Functioning, Disability and Health (ICF) model [5] shows that while the diagnosis is highly relevant, additional factors and components can impact the quality of a person’s life [6] (Figure 1). Thus, the ICF model is a useful framework for describing the functioning of patients with mental disorders [7,8]. The ICF is based on the bio-psycho-social model of health and disability and classifies functionality at different levels [6]. The first component refers to functioning and disability based on dimensions of “body functions and structures” and “activities and participation.” Two other components of the model are the dimensions of “environmental factors” and “personal factors” (Figure 1).

### The level of “health condition” and “body functions and structures” of the ICF

The level of health conditions for the ICF is dedicated to various mental and physical diagnoses provided by the International Classification of Diseases (ICD-10) [9]. Studies on the prevalence of mental disorders in the elderly have reported heterogeneous results. A systematic literature review by Riedel-Heller et al. [10] shows that a number of studies report the prevalence of affective disorders and dementia in old age. For example, estimated 1-year prevalence rates for depression – the only disorder consistently examined – range from 3% to 10% [11]. For dementia, prevalence rates are estimated between 0.6% and 3.7% for 65 to 69 year olds and 25.2% to 75% for adults 90 years old or older. However, the reported prevalence rates are extremely variable, and significant reservations have been expressed regarding the uncritical use of diagnostic interviews for adults in elderly populations. To date, little empirical data are available on the prevalence of other mental disorders in the elderly. These few studies report prevalence rates of substance-related (in particular alcohol-related) disorders in elderly people (65 years or older) between 0.5 to 3.3%; for schizophrenia, schizotypal disorders and many psychotic disorders, the rate is estimated at 10%; rates of anxiety disorders are between 4 and 14%, while somatoform disorders are estimated between 0.3 and 13% [10]. However, due to the heterogeneity of these samples, it is difficult to generalise prevalence rates for mental disorders across European countries. The few European studies conducted to date focus either on the prevalence of mental disorders in the general population of 18 to 65 year olds [12] or on specific types of disorders, e.g., affective disorders [13] or dementia [14].

Multiple studies have shown high comorbidity between mental disorders and physical illness in the elderly [13,15-17]. Studies have also shown an interaction between different physical illnesses (e.g., diabetes mellitus, cancer, cardiovascular disease) and the occurrence of depression in the elderly [13]. Adamis and Ball [15] reported a strong relationship between depression and hypertension, diabetes and cardiovascular illnesses in the elderly. In contrast, Newson [16] found no relationship between atherosclerosis and depression in the elderly. Braam et al. (2005) concluded that the link between functional disability and depressive symptoms in later life requires assessment and management of disabilities when treating depression in older populations.

### The level of “activities and participation” of the ICF

From the perspective of the ICF (see Figure 1), additional factors associated with mental and physical illness and functioning can be found to impair activities and participation in older people. Currently, studies of the degree to which older people are involved in society are underway. For example, Agahi and Parker [18] investigated the degree to which older adults participated in leisure activities over a 10 year period in Sweden. The results of that study showed that individuals in their late 70s and early 80s were better integrated in society and more likely to be involved in leisure activities if they had a higher education and exercised more. Harvey et al. [19] also showed that increased leisure-time activity was negatively associated with symptoms of depression independent of activity intensity.

### The level of environmental factors of the ICF

Environmental factors based on the ICF include material and social factors (e.g., health care services) as well as attitudes regarding the environment in which a person is living [5]. Most international studies agree that people meeting the criteria for a mental disorder are typically under recognised and untreated. Few patients receive specialist care (e.g., psychotherapy, psychotropic medication or inpatient therapy) [17,20]. This lack of treatment may cause higher health care costs (e.g., due to longer hospital stays) [12,21,22]. For this reason, it is particularly important to investigate the factors that hinder people from utilising specialist treatment. To date, only a few empirical studies have been conducted regarding the predictors of service utilisation and the barriers to utilisation faced by people who suffer from affective disorders [20,23,24] and in European people between the ages of 18 and 65 years [12]. Empirical studies have identified a fairly consistent list of factors that influence the utilisation of specialist treatment including the severity of the mental disorder [25], the perceived quality of life, the existence of a mental disorder (especially affective disorders) or multiple mental disorders, social support (e.g., family status), age (the peak of utilisation being between 35 to 49 years old) and gender [20,23,24]. Furthermore, it has been shown that as the incidence of these characteristics increases, more specialist treatment was used.

### Objectives and research questions

To date, few studies have collected data on the prevalence of mental disorders in elderly populations [26], especially taking into account parts of the ICF classification. There is also a strong need for greater standardisation of methods to improve the quality and comparability of epidemiological data in Europe.

Because the existing structured diagnostic interviews are not appropriate for the elderly, the main aim of the MentDis_ICF65+ (Mental Disorders in the elderly based on the concept of the ICF) study is 1) to adapt a structured diagnostic interview for the assessment of mental disorders in older people according to ICD-10 and DSM-IV standards. Based on this adaptation 2) prevalence rates of mental disorders in the elderly in different European and European associated countries will be assessed along with incidence rates and their relationship to symptom severity, levels of activity and participation and service utilisation as covered by the ICF dimensions.

The following research questions (RQ) can be derived:

**RQ 1**: How feasible is an adapted version of a standardised/structured diagnostic interview for the needs of people age 65 and above in different European and European associated countries?

**RQ 2**: How reliable and valid are the adapted and translated standardised/structured diagnostic interview and the newly translated instruments?

**RQ 3**: What are the point, year and lifetime prevalence rates of mental and physical disorders among the elderly population of different European and European-associated countries and what is the relationship of this prevalence to symptom severity, activities and participation and service utilisation?

**RQ 4**: What is the one-year incidence rate of mental disorders among community respondents aged 65 and above and what is the 12-month course and outcome (prognosis) of mental disorders in older people in different European and European associated countries?

## Methods/design

### Study design

**Figure 2:**
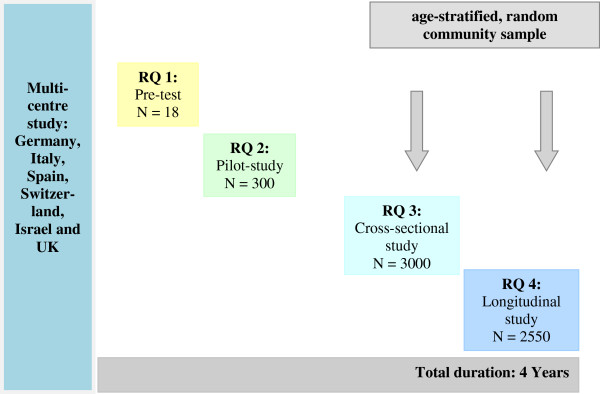
**Design of the MentDis_ICF65+ study.** Note: RQ = research question.

The study design of the MentDis_ICF65+ study is depicted in Figure 2. To answer **RQ 1**, a pre-test phase was conducted. This phase was devoted to the adaptation of age- and gender-specific assessment tools (e.g., Composite International Diagnostic Interview, CIDI) and to the translation and back translation of these instruments for older people. This step was completed in the course of preparing this project. The preparations included an in-depth review of the relevant literature followed by an elaborate multi-level analysis of the quality criteria for different assessment tools (e.g., practicability, reliability and validity), as well as language availability and prior application in an elderly sample. Following the selection of instruments, the process of adaptation was conducted. Various methods were used to test the feasibility, acceptability and usability of the adapted instruments, including expert-panel review, respondent debriefing, simple testing (interviewer feedback), and behaviour coding from a sample of 18 participants with and without mental disorders. This study was conducted at two centres in Hamburg (Germany) and London (UK) (see Figure 2).

For **RQ 2**, a pilot-phase will be undertaken. A sample of 50 inpatients and outpatients in each of 6 countries (for a total of 300 patients) will be chosen. These patients presented with different mental and physical disorders will be interviewed in Germany, Hamburg coordinating centre of the study and Dresden as well as in Italy Ferrara, Israel Jerusalem, Spain Madrid, Switzerland Geneva and in UK London. To assess instrument reliability, a subsample of 150 inpatients and outpatients (25 from each country) with different mental and physical disorders will be interviewed twice. The time interval between the interviews shall be 3 to 7 days (Figure 2).

To answer **RQ 3** and **4**, a stepwise cross-sectional and prospective longitudinal design will be conducted. The multi-centre study in six European countries and associated states will be based on an age-stratified, random sample of 3000 subjects living in selected catchment community areas of each participating country (500 subjects from each country). Additionally, all participants in the cross-sectional study will be contacted for follow-up participation one year later. Based on prior experience from other studies, a response rate of 85% can be expected within 1 year. Thus the expected sample size is 425 elderly people in each of 6 countries for a total sample size of 2550 individuals.

### Study participants

To answer **RQ 1**, 18 participants from two study centres (London, UK and Hamburg, Germany) were interviewed with the adapted instrument. Audio files from the interviews were recorded. Each site interviewed a heterogeneous sample, equally distributed across two age groups (65-74 and 75-85 years), both with and without mental disorders.

Within the study pilot-phase (**RQ 2**), a sample of 50 inpatients and outpatients from each of the 6 participating countries (for a total of 300 patients) with different mental or physical disorders will be interviewed. The exclusion criteria will be severe cognitive impairments (MMSE cut-off score > 27), insufficient level of cor-responding language, and being younger than 65 or older than 85 years of age.

Participant inclusion criteria **RQ 3** and **4** are the ability to provide informed consent, living at home in the predefined catchment area at the beginning of the cross-sectional study, and ages between 65 and 85 years, respectively. The exclusion criteria for the participants will be severe cognitive impairment as assessed with the MMSE (Mini-Mental State Examination, Mini cut-off score > 27) [27] in the screening part of the diagnostic instrument, making the administration of assessment instruments impossible, and insufficient understanding of the corresponding language. Qualified and trained interviewers will screen patients for inclusion and exclusion criteria. These interviewers will remain in close contact with the study’s research staff.

### Ethics and quality standards

The study was approved by research ethics committees in all six centres (Germany: Hamburg Ethic committee of the Medical Association No. 2895, Italy: Ferrara No. 0096637 5/11/2009, Israel: Jerusalem No. 0376-09-HMO, Spain: Madrid No. 22032010, Switzerland: University Hospitals of Geneva ethics committee, Protocol No 09-121 and UK: National Research Ethics Service No. 10/H0715/21).

The study centre in Hamburg, Germany, is responsible for coordinating the study and for ensuring the accuracy, quality, and input of the data collected. To complete the cross-sectional and longitudinal components of the study (**RQ 3** and **4**), interviewers must be trained to administer the adapted version of the instruments. Research staff from each study centre of the Ment-Dis_ICF65+ project will be trained during a 1-2 day workshop provided by the Dresden centre under the direction of Hans-Ulrich Wittchen. Afterwards, the research staff of each centre will train up to 20 interviewers per centre on procedures for conducting the adapted interview. To ensure a high level of interview standardisation between the different centres, interviewers will be provided with protocols and guidelines. The protocols will establish procedures for opening the interview, evaluating difficult respondent questions, and for stopping the interview. These rules will be established by the study’s coordinating centre. Interviewers will be monitored and supervised continuously throughout the course of the study.

### Study measures

Table 1 gives an overview of the core study measures for the MentDis_ICF65+ project covering all domains of the ICF (see also Figure 1). These instruments will be used to answer **RQ 3** and **4**.

**Table 1:** MentDis_ICF65+ assessment battery covering ICF domains

**ICF domain: personal factors**	
**Instrument**	content
**Adapted version of the Composite International Diagnostic Interview for the elderly (CIDI65+, Wittchen et al., in preparation)**	sociodemography
**ICF domain: health condition and body functions and structures**	
**Instrument**	content
**Adapted version of the Composite International Diagnostic Interview for the elderly (CIDI65+, Wittchen et al., in preparation)**	screening for nicotine abuse
	somatic, somatoform disorders
	anxiety disorders (including HADS)
	depressive disorders
	bipolar disorders
	psychotic symptoms
	screening for alcohol abuse (including AUDIT)
	obsessive compulsive disorders
	screening for drugs and medication abuse
	posttraumatic stress disorder, adjustment disorder
	cognitive impairment (MMSE)
**Health of the Nation Outcome Scales65+ HoNOS-65+, **[28]	symptom severity
**10-item version of the Big Five Inventory BFI-10, **[29]	personality assessment
**Shalev’s Coping Efficacy Scale (CES)**	assessment of coping efficacy
**ICF domain: activities and participation**	
**WHOQoL-BREF **[30]	quality of life
**WHODAS II **[31]	Assessment of activities and participation
**ICF domain: environmental factors**	
**service utilisation**	items on access to and barriers of service utilisation

#### The Composite International Diagnostic Interview for the elderly (CIDI65+)

The core instrument of the study is a standardised diagnostic interview used to collect data on the prevalence and incidence of mental disorders in the elderly (**RQ 3** and **4**). The study group decided to adapt the widely used “Composite International Diagnostic Interview” [32] to the needs of the elderly. The study group developed the CIDI65+ under the direction of the Dresden centre (H.-U. Wittchen). The CIDI65+ is adapted to the particular social, cognitive and psychological abilities and needs of the elderly. The interview evaluates somatic morbidity, somatoform disorders, anxiety disorders (panic, panic disorder, GAD, agoraphobia, social and specific phobias), depressive disorders, bipolar disorders, psychotic symptoms, obsessive-compulsive disorders, substance abuse (screening sections for nicotine, alcohol, drugs/medication), adjustment disorders, acute stress- and post-traumatic stress disorders as well as cognitive impairment. The adapted version of the CIDI will be translated into all required languages.

#### The Health of the Nation Outcome Scales65+ (HoNOS65+)

Originally, the Health of the Nation Outcome Scales (HoNOS) were developed for the routine assessment of adults with mental disorders [33]. Additional instruments, such as the HoNOS65+, have been developed to augment the “HoNOS family”. The HoNOS65+ is an instrument for assessing the severity level of 12 problem areas (e.g., item 7: depressive mood; item 2: self-harm). These areas are assessed on a scale from 0 (no problem) to 4 (severe to very severe problem). A glossary containing anchor examples for the allocation of individual ratings (from 0 to 4) is available for the 12 problem areas. Numerous studies have assessed the mostly satisfactory psychometric properties of the HoNOS65+ [34]. Most of these studies have been conducted in Great Britain and Australia where this instrument is being used as part of the national health strategy as a “Minimum Data Set.” Moreover, the HoNOS65+ is one of the six most commonly used assessment scales in elderly psychiatry services, providing key material results for shaping the provision of psychiatric services for older individuals [35].

#### The short version of the Big Five Inventory (BFI-10)

To further assess personality traits as another important component of the body function and structure domain based on the ICF, we will use the short version of the Big Five Inventory (BFI-10). This instrument was developed from the 44-item version of the BFI by Rammstedt and John [29]. The BFI-10 covers five personality domains: extraversion, agreeableness, conscientiousness, neuroticism, and openness on a five-point Likert scale (1 = disagree strongly to 5 = agree strongly). This shortened version of the BFI provides satisfactory psychometric properties [29]. So far, the few existing studies on the relationship between personality and mental disorders in the elderly argue in favour of a dimensional approach of personality in late life [36].

#### World Health Organization’s QOL measure (WHOQoL-BREF)

To measure the quality of life in mentally ill older persons, the WHO Quality of Life short version [WHOQoL-BREF, 30] will be used. The WHOQoL-BREF was developed by the World Health Organization [30] from the WHOQoL-100 item version as a 26-item questionnaire and uses a five-point Likert scale to assesses the individual’s perceptions in the context of their culture and value systems, and their personal goals, standards and concerns. The questionnaire measures dimensions including physical and psychological well-being, environmental factors and social support. The psychometric properties were found to be satisfactory [37]. Moreover, there is evidence, that the WHOQoL-BREF can be successfully administered in older people [38].

#### World Health Organization disability assessment schedule II (WHODAS II)

An assessment of activities and participation (based on ICF categories) will be performed using the World Health Organization Disability Assessment Schedule II (WHODAS II) [31]. The WHODAS II is a generic instrument assessing the functional impairment of daily activities in six different areas (including communication and self-supply). An extended 36-item expert rating version and a 12-item self-rating short version are available. A study by Pösl [39] applied the extended version (36 items) of the WHODAS II as a self-rating instrument. Satisfactory psychometric scores for patients with affective disorders were reported regarding reliability and validity. A study by Kim et al. [40] used the WHODAS II in a sample of people aged 65 years and older. In this study, the level of impairment measured by the WHODAS II was more influenced by somatic health, depression and cognitive functioning than by sociodemographic factors. This study provided the first evidence that the WHODAS II is an adequate instrument for the assessment of these impairments in old age.

#### Shalev’s coping efficacy questionnaire (CES)

This short rating scale consists of four items addressing domains of coping efficacy: ability to pursue task performance, emotional control, ability to sustain rewarding interpersonal contacts and ability to maintain positive self-image.

The corresponding instruments (see above) will be translated according to the criteria of the WHO [41], if they are not yet available in each country. Following a rating by independent experts and subsequent verification, the instruments will be retranslated by a native speaker blind to the translated version. Test authors will then evaluate possible divergences in the retranslations and work to resolve inconsistencies before incorporating these changes into the translated version. This procedure will be repeated until a consensus between original and translated versions has been reached. Semantic, content and technical correspondence will be monitored consistently [42].

### Statistical methods

#### Power calculation

The project pre-test (**RQ 1**) was used to identify feasibility issues when using the adapted instruments. Therefore, no power calculation is necessary.

The pilot-phase (**RQ 2**) of the study will be undertaken to analyse the psychometric properties of the adapted version of the CIDI65+ and the newly translated instruments. A sample size of 300 participants would provide 80% power to detect a medium effect size (r = 0.30) according to Cohen [43], using a two-tailed test at a significance level of <0.05.

The required sample size for answering **RQ3** and **RQ4** was calculated using an expected prevalence rate of 30%, based on reported lifetime prevalence rates of mental disorders from all age groups and countries. The expected standard errors/widths of the 95% confidence intervals for each prevalence estimate will be as follows (see Table 2):

**Table 2:** Sample size calculation for the cross-sectional and longitudinal study

	**Expected no. of evaluable patients**	**Expected standard error**	**Expected width of 95% confidence interval**
Overall	3,000	0.8%	± 1.7%
Country	500	2.0%	± 4.1%
5-years age group	1,000	1.4%	± 2.9%
5-years age group per country	167	3.5%	± 7.1%

The minimum difference in prevalence rates between two pre-specified countries that can be detected with a power of 80% or 90% is 9.2% (from 34.6 to 25.4%, risk reduction 32.0%) or 9.4% (from 34.7 to 25.3%, risk reduction 36.2%).

#### Sampling and stratification (RQ 3 and 4)

Administrative differences in each country may require the use of multiple sampling strategies. Samples will be collected using either a register of residents or postal residents (most centres), telephone numbers or a register of primary care patients. Reasons for non-participation and non-eligibility (e.g., cognitive impairment, problems understanding the language) will be documented. In addition, we will collect information about non-responders (e.g., age and gender) to examine any selective attrition effects.

Each study centre will define a catchment area prior to recruitment. This catchment should be representative of the region’s population with regard to social classes (equal inclusion of areas with working, middle and upper social class population), specific living conditions of our age cohort (e.g., no exclusion of nursing homes) and population structure (urban/rural). The main aim of this approach is to estimate the centre-specific and the overall prevalence of mental disorders in 65 to 85 year olds as precisely as possible. To maximise estimator accuracy, some strata may be oversampled. Therefore, all the strata should be filled equally to reach equal variances for equal proportions (see Table 3).

**Table 3:** Stratification sample of the MentDis_ICF65+ study

**Age/gender**	**65 - < 75**	**75 - < 85**	**Total**
Male	125	125	250
Female	125	125	250
Total	250	250	500

The resulting cohort will be drawn according to the stratification criteria (Table 3). Each study centre will draw a sample of at least 2000 persons based on an expected response rate of 20 to 25%. All cross-sectional study participants will be asked to participate again one year later. Based on previous study experience, a 1-year response rate of 85% can be expected. Thus, the study aspires to collect a sample of 425 elderly individuals in each country for a total sample size of 2550.

#### Statistical analyses

To answer **RQ 1**, a feasibility analysis on the adapted instrument will be undertaken. This analysis will employ methods including cognitive testing and behaviour coding.

To test **RQ 2**, analyses of the psychometric properties of the CIDI65+ and the newly translated instruments (e.g., German version of the HoNOS65+) will be conducted. Kappa statistics will be calculated to examine test-retest reliability of the CIDI65+. Kappa values below 0.40 will be considered to have poor agreement, values between 0.40 and 0.60 will have fair agreement, values between 0.61 and 0.75 will be in good agreement and values of 0.76 and above will have excellent agreement [44]. Because sufficient sample sizes are required, coefficients will only be calculated for those diagnoses with a base rate of at least 10% (i.e., at least 15 cases diagnosed in either the test or the retest interview). Agreement within the continuous variables will be measured using the Intraclass-Correlation-Coefficient (ICC). The ICC will be calculated for variables of the CIDI65+, including age of onset and duration of the disorder. Furthermore, the newly translated instruments in each country will be analysed for reliability (e.g., internal consistencies) and validity (e.g., criterion validity).

As the study aims to examine the prevalence and incidence rates of mental disorders in the elderly of all six countries and associated states (**RQ 3** and **4**), the data from all the participating centres will be analysed in a comprehensive statistical model. This model will provide an estimate of 1) the global prevalence and 2) the 1-year incidence of mental disorders. Mixed generalised linear models, with country as a random effect and stratum (age*sex) as a fixed effect, will be used to estimate prevalence and incidence. In addition, the BLUP estimators returned by the model will be used to analyse differences between the participating centres. For all estimators, the corresponding confidence intervals will be denoted. The estimated prevalence rates will be projected to the total population of the participating countries based on the publicly available population statistics to give an impression of the absolute number of elderly cases requiring health care. These prevalence and incidence estimations for mental disorders (point, 1-year and lifetime prevalence) in older people in absolute figures, and 95% confidence interval and predictors of mental disorders on the basis of binary logistic regression analyses (including affective disorders as a variable criteria; and including age, gender, education, living situation, and partner situation as independent variables) should also be calculated. Furthermore calculation of odds ratios and confidence intervals, comorbidity rates and correlations to physical disorders should be performed. Regression analyses will be used to predict levels of activities and participation (including mental disorders, physical disorders or other independent variable) and quality of life. Analyses using binary logistic regression analyses (utilisation of professional services as a dependent variable and the aforementioned independent variables) and calculation of odds ratios and confidence intervals will also be performed. In addition, data from non-responders will be analysed to identify differences between responders and non-responders.

## Discussion

Due to the current state of the literature, thus far, no empirical data exist that are based on a sufficiently detailed and sound methodological assessment of mental disorders in elderly people (65+) from different countries in Europe and associated states. The aim of the proposed study is to establish a common methodology. Finally, the results of this population from this Europe-wide project will, beyond the mentioned epidemiological aims, provide vital information on professional service provision as well as information on the utilisation of relevant data for policy-making decisions.

The results of the study will contribute to the following research areas:

– The adaptation and translation of instruments for the assessment of mental and physical disorders in older people (e.g., CIDI65+, WHODAS-II, HoNOS-65+)

– Establishing psychometric properties of and harmonising instruments for application within cross-sectional samples

– Establishing prevalence rates (point, year and lifetime) of mental and physical disorders in the elderly in different countries of the EU

– Identifying the level of mental disorder severity in older people

– Identifying factors that promote and impair access to health services

– Establishing levels of activity and impairment (based on ICF concepts) in older people with mental and physical disorders

– Rating the quality of life in older people with mental and physical disorders

– Quantifying the one-year incidence rate of mental disorders and the 12-month course and outcome of mental disorders in older people in different European countries

– Identifying predictors affecting the course of mental and physical disorders of the elderly in the general population of six different EU countries

For the first time, data on physical and mental illnesses, their related problems and the need for support will be collected. In addition, valuable instructions for daily health care can be derived from the instruments developed for the elderly population. This project considerably extends the current level of knowledge. Furthermore, the longitudinal design of the Ment-Dis_ICF65+ study allows for the assessment of mental disorder incidence rates in the elderly, providing new data that can be used for health policy and care planning.

## Competing interests

The authors declare that they have no competing interests.

## Authors’ contribution

The authors collectively drafted the study protocol and approved the final manuscript. SA is the guarantor. All authors read and approved the final manuscript.

## Pre-publication history

The pre-publication history for this paper can be accessed here:

http://www.biomedcentral.com/1471-244X/13/62/prepub
